# Do refinements to original designs improve outcome of total knee replacement? A retrospective cohort study

**DOI:** 10.1186/1749-799X-9-7

**Published:** 2014-02-06

**Authors:** Marieke J Piepers, Ruud P van Hove, Michel PJ van den Bekerom, Peter A Nolte

**Affiliations:** 1 Department of Orthopaedics, Spaarne Hospital, Spaarnepoort 1, Hoofddorp, 2134 TM, the Netherlands; 2 Department of Orthopaedics, O.L.V.G, Oosterpark 9, Amsterdam, 1090 HM, the Netherlands

**Keywords:** Total knee replacement, Design change, Clinical outcome, Survival, Low contact stress

## Abstract

**Background:**

Long-term results of the 'classic’ low contact stress (LCS) total knee replacement (TKR) have been satisfactory; nonetheless, design changes have been made which resulted in the 'complete’ LCS TKR. The aim of this study is to compare the 5-year incidence of revision and midterm clinical performance before and after introduction of the 'complete’.

**Methods:**

A retrospective cohort analysis was conducted on 100 primary uncemented TKRs of both designs. At 5-year follow-up, revision and reoperation rates were determined for these 200 TKRs. Knee Society score (KSS), the Oxford Knee score (OKS) and range of motion were determined for 143 TKRs.

**Results:**

In the 'classic’ cohort, 3% of the TKRs were revised compared with 5% in the 'complete’ cohort (*p* = 0.72).The mean KSS was 134.1 (SD 38.3) in the 'classic’ cohort compared to 135.0 (SD 42.8) in the 'complete’ cohort (*p* = 0.89). Of the 'complete’ TKRs, 35.2% scored within the lowest quartile of the KSS knee compared to 16.7% of the 'classic’ TKRs (*p* = 0.01). The OKS was 23.3 (SD 9.3) in the 'classic’ cohort compared to 22.5 (SD 10.1) in the 'complete’ cohort (*p* = 0.45). More than 5° flexion contracture was only found in four patients in the 'complete’ cohort (*p* = 0.04).

**Conclusions:**

No statistical difference in revision rate and average scores for midterm clinical performance was observed between the 'classic’ and the 'complete’. However, the 'complete’ cohort had a higher percentage of KSS Knee in the lowest quartile, which suggests a clinical relevant difference compared with the 'classic’. Further investigation in future studies with new designs is needed.

## Introduction

The low contact stress (LCS, DePuy, Warsaw, Indiana, USA) 'classic’ mobile-bearing total knee replacement (TKR) was introduced more than three decades ago. This mobile-bearing TKR consists of a cobalt–chromium–molybdenum femoral and tibial component with gas plasma sterilized GUR® 1050 ultra-high molecular weight polyethylene (UHMWPE) insert. The insert is a rotating platform and allows rotation between the insert and the tibial component. The intention of the dual surface articulation is to reduce stress at the bearing surfaces and bone implant surfaces by maximizing conformity of the tibial and femoral components [1]. Excellent results were obtained with mobile-bearing TKR, with only 4.5% failures and 96.4% implant survivorship at 15-year follow-up [2].

**Figure 1:**
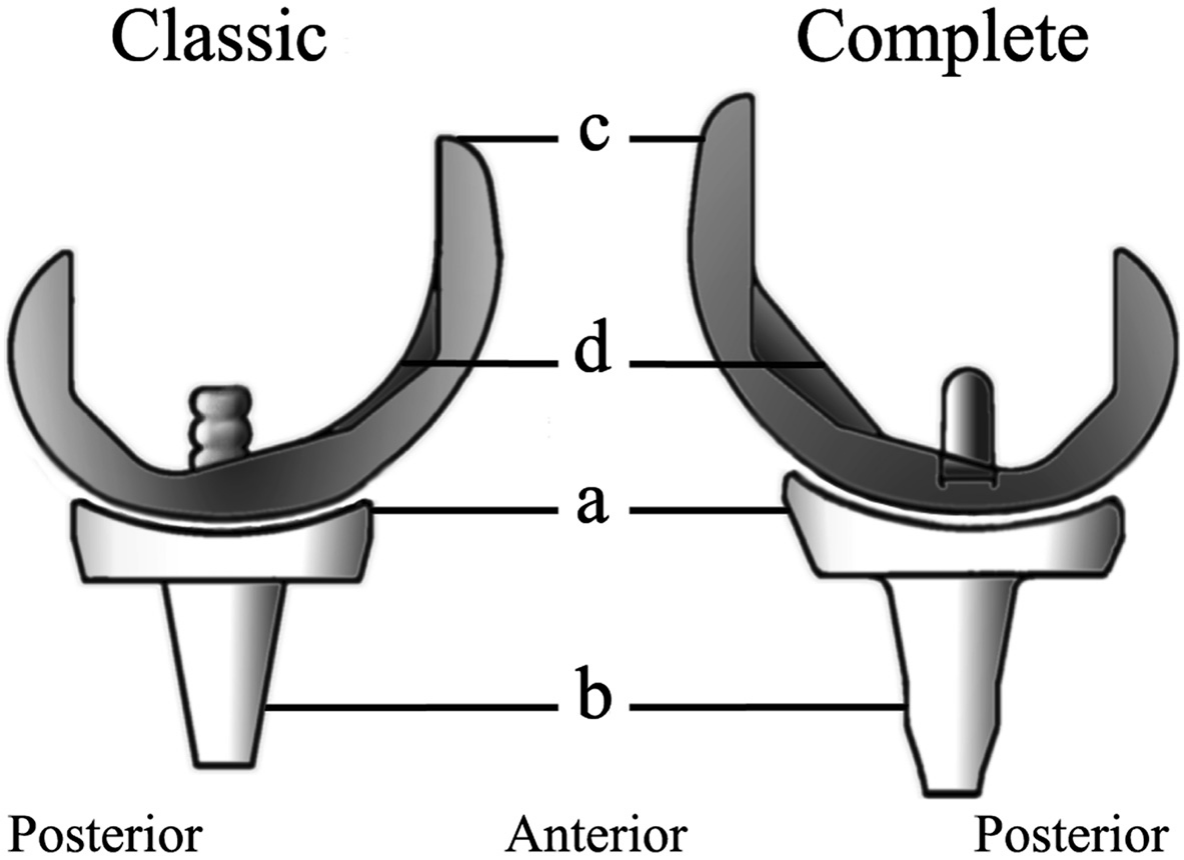
**Lateral view of the 'classic’ and 'complete’ insert and femoral component. (a)** Higher anterior lip of the insert of the 'complete’ compared with the 'classic’. **(b)** Cylindrical segment at the 'complete’ insert compared with the previously tapered 'classic’ insert stem. **(c)** Extended anterior flange of the 'complete’ femoral component compared with the anterior flange of the 'classic’ femoral component. **(d)** Flat interior surface of the 'complete’ femoral component compared with the curved interior surface of the 'classic’ femoral component.

**Figure 2:**
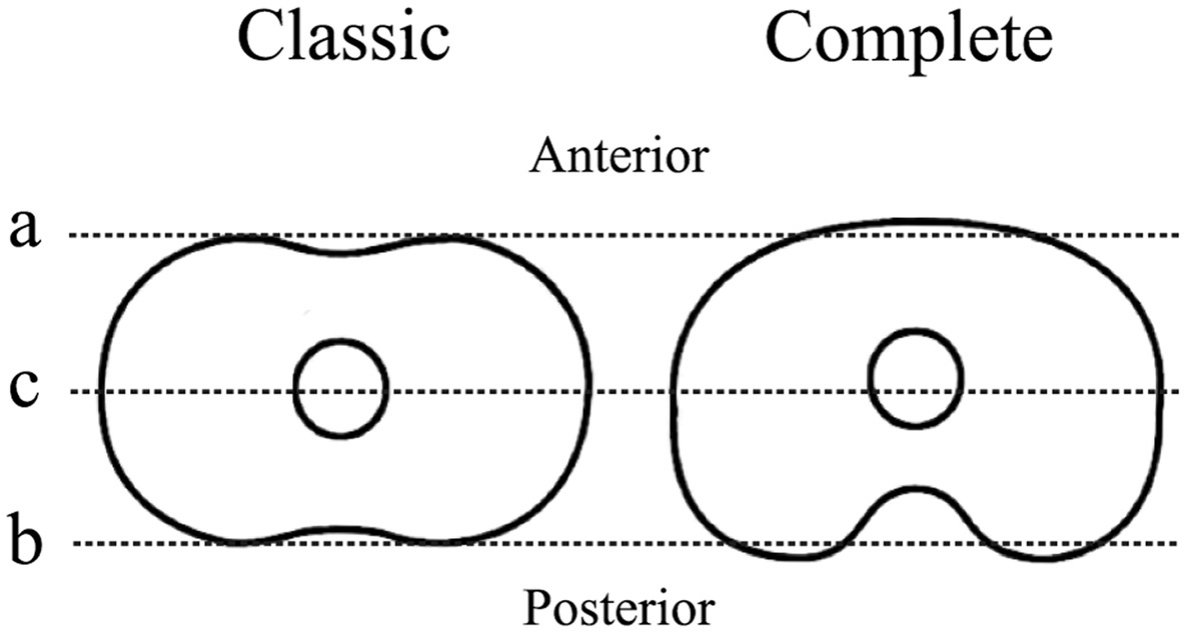
**Transverse view of the 'classic’ and 'complete’ tibial tray. (a)** The difference in outline; anteriorly, the contour was straightened out. **(b)** Posteriorly, the indentation is more prominent in the 'complete’. **(c)** Rotation point of insert is slightly ventrally positioned in the 'complete’.

In 1985, the LCS 'universal’ succeeded the 'classic’. The insert of the 'classic’ gave rise to the probability of insert dislocation [3,4]. Resistance to dislocation of the insert is determined by the contact pressure and engagement depth of the femoral component in the insert [5]. In order to improve resistance to dislocation and to facilitate insertion of the insert, several modifications were made to the insert of the 'universal’. In 2001, the 'complete’ was introduced with further modifications. The insert of the 'complete’ is made from a gamma irradiation vacuum in foil sterilized GUR® 1020 UHMWPE. The anterior lip of the insert was raised, and the posterior lip was lowered (Figure 1, part a). A cylindrical segment was added to the distal part of the previously completely tapered insert stem (Figure 1, part b). The tibial component was modified to increase the coverage of the proximal tibia. The dimensions in anterior posterior (AP) direction were increased (Figure 2), the concave anterior contour was straightened out (Figure 2, part a) and the posterior concave indentation was made more prominent (Figure 2, part b) [6]. The central anchor peg of the tibial component was moved 2.5 mm ventrally (Figure 2, part c). The anterior flange of the femoral component was narrowed on both sides and was extended for several millimetres (Figure 1, part c). To aid femoral component removal, disimpaction slots were added and the fixation pegs were smoothed out. To simplify resection of the femoral groove, the curved shape of the interior surface of the femoral component was replaced by a flat surface (Figure 1, part d). Finally, the depths of the graft pockets were slightly reduced to optimize the fixation interfaces. Geometry of the articulating surfaces remained unchanged for all versions of the LCS TKR.

The 'complete’ was introduced in our hospital in September 2005 because the 'classic’ became unavailable. The 'universal’ was not introduced in our hospital because of satisfactory results and a low incidence of insert dislocation in our hospital with the 'classic’. With the introduction of the 'complete’, some of the instruments used for tibial preparation were also replaced. Clinical observation suggested a higher revision rate with the 'complete’ due to loosening of the tibial component. To establish this clinical observation, we performed a retrospective study with primary outcome revision rate and secondary outcome clinical midterm results of the 'classic’ compared with the 'complete’ at 5 years.

## Methods

### Patient selection

The study involved a retrospective cohort analysis of 200 TKRs in 184 patients to evaluate the postoperative outcome after the change from 'classic’ to 'complete’ TKR. Patients were reversibly anonymized, and data from their medical files concerning TKR were collected. Patients were contacted by phone and asked to attend the 5-year TKR follow-up and to participate in this study. After verbal consent, patients were seen at the outpatient clinic. After consulting the local medical ethical committee, the need for ethical clearance of this study was not warranted.

Six patients of the study group received the 'classic’ bilaterally, and seven patients received the 'complete’ bilaterally. Three patients had bilaterally TKR with the 'classic’ as well as the 'complete’. The 'classic’ cohort included 100 TKRs consecutively performed before the introduction of the 'complete’ between February 2005 and September 2005. The 'complete’ cohort included 100 consecutive uncemented primary TKRs performed from September 2005 to March 2006.

Revision and reoperation rate, as well as reason for revision or reoperation was available for 200 TKRs. Clinical evaluation after surgery could not take place in 31 TKRs because of death of the patient that was not related to knee surgery (23 TKRs) and revision (8 TKRs). Of the remaining 169 TKRs, 143 TKRs (84.6%) were clinically evaluated of which 72 in the 'classic’ cohort and 71 in the 'complete’ cohort. In 26 TKRs (15.4%), patients were not physically or mentally able to participate in this study. Three patients were unwilling to give consent, two with a 'classic’ TKR and one with a 'complete’. There was no difference in patients lost to follow-up between the two cohorts.

### Surgical technique and postoperative management

Cementless TKR was performed using a medial parapatellar incision and a tibia cut as the first approach. For tibial preparation, a tibial reamer was used for the 'complete’ and a tibial centering punch was used for the 'classic’.

Surgery was performed either by one of three knee surgeons or by a specialist registrar under direct supervision of one of these knee surgeons. Postoperative management for TKR was standardized according to protocol. No changes were made to this protocol between February 2005 and March 2006. Deep vein thrombosis prophylaxis, antibiotic and analgetic regimen, postoperative nursing care and a rehabilitation programme were identical for the two cohorts.

### Outcome assessment

For information on revision or reoperation, available medical records, information retrieved from the patient and/or first line relatives were used. We defined revision as surgical removal or replacement of parts of the prosthesis, for all reasons other than infection. A reoperation was defined as an intraarticular intervention, i.e. arthroscopy, arthrotomy. The focus of this retrospective review is to compare the effectiveness of the TKR design as such. There was no difference in infection rates and related reoperations, but these were not included in this review.

Five years or more after the index surgery, a single unblinded examiner, who was not involved with the initial operation, clinically evaluated the patients. Range of motion and alignment were assessed using a goniometer. The Knee Society score (KSS) and the Oxford Knee score (OKS) were assessed.

The KSS consists of a Knee score and functional score. The KSS knee (Insall modification, 1993) consists of three items: experienced pain (50 points), knee stability (25 points) and range of motion (one point for each 8°). A reduction in the score is a result of extension lag, flexion contracture, malalignment and pain experienced at rest, giving a maximum deduction of 70 points. The KSS function rates the achievable walking distance (50 points) and climbing the stairs (50 points). Points are deducted if an aid is needed, to a maximum of 20 points. The lowest score is zero points [7].

The OKS consists of 12 questions addressing pain and mobility. Each of the 12 questions receives a score on a scale of 1 to 5 points. The lowest score of 12 points indicates no pain and little restrictions to mobility, and a score of 60 points indicates severe limitations to mobility and the presence of pain [8,9].

Pain is considered to be the true representation of the success of a TKR [10]. Therefore, additional questions from the Algofunctional Index were included addressing pain experienced during specific normal day activities [11]. These items were an integral part of the evaluation including the KSS and OKS.

To find a possible learning curve after the introduction of the 'complete’, revisions as well as the KSS Knee of the TKRs in the 'classic’ and the 'complete’ cohort performed by the senior author (PN), one of the three knee surgeons, were scored.

The follow-up period for the 'classic’ cohort was 69 months (mean, range 62–75) and 64 months (mean, range 62–67) for the 'complete’ cohort (*p* < 0.001).

### Statistical methods

Data collection was performed using Microsoft Access 2007 Software (Microsoft Corporation, Redmond, Washington, USA). Data analysis was done using SPSS 20.0 statistical software (IBM SPSS, New York, USA). For further analysis of the KSS Knee, score ranges were created in which 25% of all clinically evaluated patients scored, resulting in quartiles. The number of patients per cohort in each quartile was compared. Comparisons between the results of the KSS, OKS, knee range of motion and time intervals were made using the independent sample *t* test. If variances were not equal, computed with Levene's test, Welch's *t* test was used. The Pearson's chi-square test was used to compare the revision rates between both cohorts. Individual questions were further analysed using the Mann–Whitney U rank–sum test. The Kolmogorov–Smirnov test was used to assess normal distribution. *Post hoc* power analysis was performed to interpret and explain the observed data as proposed by Hoenig and Heisey [12]. Differences were considered statistically significant when *p* < 0.05.

## Results

Of all 200 TKRs, 148 were performed in female patients and 52 in male patients. The patient's average age at the time of the index surgery was 71 years (mean, range 46–93 years). The mean body mass index was 28.5 kg/m^2^ (range 19.3–46.3 kg/m^2^). The American Society of Anaesthesiologists (ASA) classification I was assigned 42 of the TKRs, ASA II in 145 knees and ASA III in 23 TKRs. The underlying diagnosis was primary osteoarthritis in 182 of the 200 TKRs (91%), rheumatoid arthritis in 13 TKRs (6.5%) and posttraumatic arthritis in 5 TKRs (2.5%). There was a higher rate of patients with rheumatoid arthritis in the 'classic’ cohort (*p* = 0.048). Eleven patients had previous high tibial osteotomy of which ten were followed by removal of osteosynthesis material prior to the index surgery. One patient underwent one stage removal and TKR. No other types of osteosynthesis were performed prior to TKR. There was no other difference in baseline characteristics between both cohorts (Table 1). The total cohort was compared with the cohort available at follow-up, the average BMI was higher (*p* < 0.001) in the cohort available at follow-up.

**Table 1:** Patient characteristics

	**All TKRs**			**Clinically evaluated TKRs**		
	**Classic (*n*** **= 100)**	**Complete (*n*** **= 100)**	***p *** **value**	**Classic (*n*** **= 72)**	**Complete (*n*** **= 71)**	***p *** **value**
Age (years (SD))	71.3 (6.8)	70.8 (8.5)	0.67	70.5 (7.9)	69.9 (7.3)	0.62
Male (%)	34	36	0.77	35	42	0.36
BMI (kg/m^2^ (SD))	28.5 (4.9)	28.6 (4.7)	0.94	29.5 (5.0)	29.0 (4.2)	0.51
Right (%)	59	58	0.89	54	58	0.67
Underlying diagnosis (%)			0.12			0.35
Osteoarthritis	87	95		87.5	94	
Posttraumatic arthritis	3	2		4.2	1.4	
Rheumatoid arthritis	10	3		8.3	4.2	
High tibial osteotomy (%)			0.49			0.47
Varus deformity	6	4		6.9	4.2	
Valgus deformity	1	0		1.4	0	
ASA (%)			0.98			1.00
1	21	21		23.6	23.9	
2	68	67		66.7	66.2	
3	11	12		9.7	9.9	
Surgeon (%)			0.76			0.44
1	37	42		33.3	43.6	
2	41	37		44.4	36.6	
3	22	21		22.2	19.7	

BMI, body mass index; ASA, American Society of Anaesthesiologists classification.

In the 'classic’ cohort, there were 11 TKRs in patients who died of causes not related to knee surgery compared to 12 TKRs in the 'complete’ cohort (*p* = 1.00). The mean time to death in the 'classic’ cohort was 42.6 months (SD 18.8) compared to 35.4 months (SD 18.5) in the 'complete’ cohort (*p* = 0.37). There were no reoperations in the group of TKRs of patients who died of causes not related to knee surgery.

### Revisions and reoperations

In the 'classic’ cohort, three (3%) of the TKRs were revised. In two cases, loosening of the tibial tray was indicated, and in a single case, assumed hypersensitivity to the implant material resulted in revision surgery. In the 'complete’ cohort, five (5%) of the TKRs needed revision; in four cases, the indication was loosening of the tibial tray, and in one case, loosening of the femoral component. *Post hoc* power of this observation was 10.8%. Revision was preceded by arthroscopy in one 'classic’ and one 'complete’ TKR (25%) and by mobilization under anaesthesia (MUA) in two 'classic’ TKRs (25%). No differences in incidence of revision (*p* = 0.72), reoperations (*p* = 1.00) and limited function requiring MUA (*p* = 0.44) were found between both cohorts (Table 2).

**Table 2:** Revision and reoperation rates

	**Classic (*n*** **= 100)**		**Complete (*n*** **= 100)**		***p *** **value**	
	***n***	**Int**	***n***	**Int**	***n***	**Int**
Revision	3	32 ±31.7	5	14 ±4.6	0.72	0.43
Loosening of tibial tray	2	14 ±5.0	4	15 ±4.2	0.68	0.72
Loosening femoral component	0		1	9		
Hypersensitivity to implant	1	68	0			
Reoperation	8		9		1.00	
Arthroscopy	7	15 ±8.3	7	8 ±5.4	1.00	0.07
Arthrotomy	1	27	1	0	1.00	
Patellar replacement	1	25	1	12	1.00	
MUA	5	2.6 ±1.3	2	2.5 ±0.7	0.44	0.52

Values are given as mean ± standard deviation. Int, interval, time from index surgery to intervention in months; MUA, mobilization under anaesthesia.

The indications for reoperation and MUA were stiffness, pain and/or persistent swelling of the knee. Reoperations were performed in eight (8%) of the 'classic’ cohort and nine (9%) of the 'complete’ cohort. Two reoperations were performed on one patient with a 'classic’ TKR, an arthroscopy followed by an arthrotomy. One week after implantation, a 'complete’ TKR needed open reposition because of malposition of the tibial tray. MUA because of stiffness was performed in five patients in the 'classic’ cohort and two in the 'complete’ cohort (*p* = 0.44) (Table 2). No difference was observed in time to revision (*p* = 0.43) or reoperation, i.e. arthroscopy (*p* = 0.07), arthrotomy (*p* = 1.00), patellar replacement (*p* = 1.00) (Table 2).

In the 'classic’ cohort, six patients had a superficial wound infection compared to seven in the 'complete’ cohort (*p* = 1.00). Deep infection within 3 months after surgery occurred in two patients in the 'classic’ cohort and one patient in the 'complete’ cohort (*p* = 1.00). In both cohorts, deep infection occurred in one patient more than 3 months after surgery (*p* = 1.00). These patients were treated successfully by open or arthroscopic irrigation, debridement, component retention and intravenous antibiotics and were not excluded for clinical evaluation.

### Clinical evaluation at follow-up

**Figure 3:**
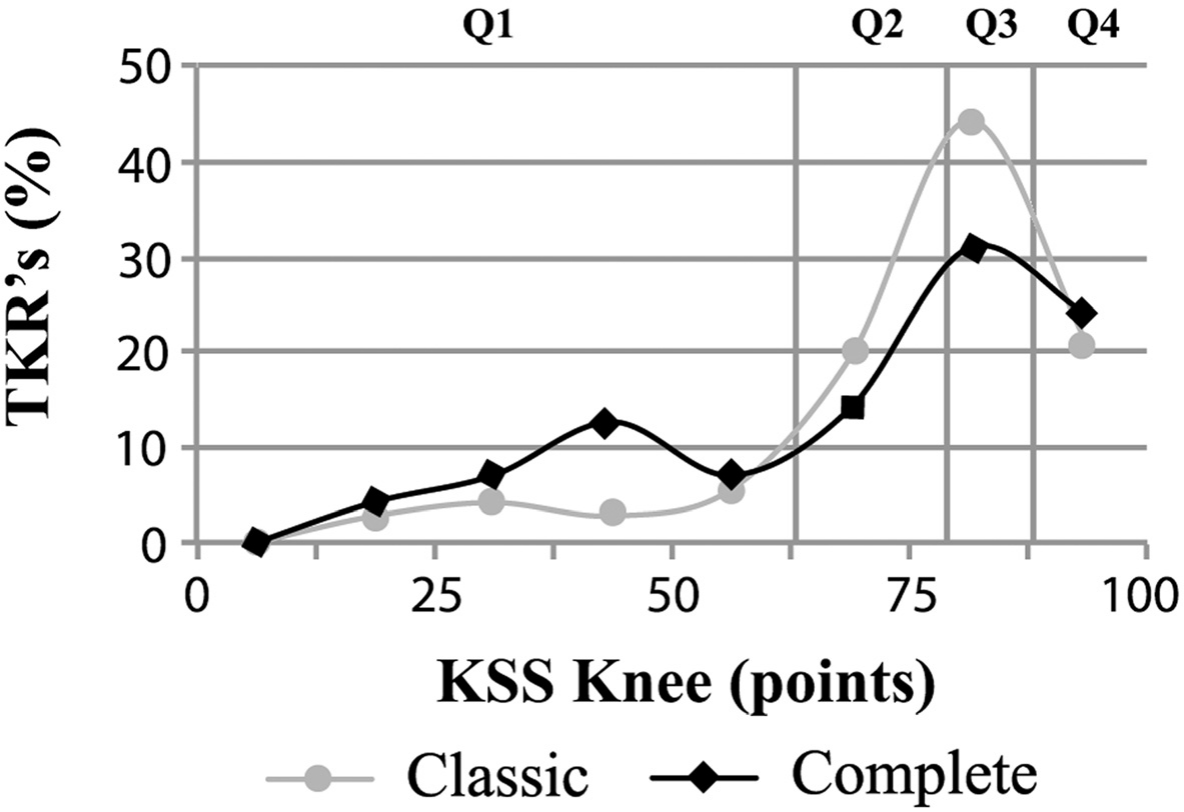
**KSS Knee distribution for 'classic’ and 'complete’ TKR.** The *Y*-axis represents the percentage of TKRs of the 'complete’ and 'classic’ cohort. A higher percentage of patients in the 'complete’ cohort is found in the lowest quartile. More specific, 13% of the 'complete’ TKRs score between 37.5 and 50 points compared with 3% of the 'classic’ TKRs. Q1, lowest quartile; Q2, second quartile; Q3, third quartile; Q4, highest quartile.

Neither design showed a clinical advantage based on the mean KSS, OKS, flexion and extension (Table 3). The KSS Knee and function as well as the OKS were not normally distributed. We used quartiles to show the distribution of the KSS Knee. Quartiles consisted of a range of KSS Knee in which 25% of all clinically evaluated patients scored and the following KSS Knee ranges were created: lowest quartile KSS Knee 0–63, second quartile KSS Knee 64–79, third quartile KSS Knee 80–87 and highest quartile 88–100. In the 'classic’ cohort, 12 (16.7%) TKRs were found in the lowest quartile compared to 25 (35.2%) of the 'complete’ cohort (*p* = 0.01) (Figure 3). A marked difference is found in the number of patients scoring between 37.5 and 50 points: 2 (3%) of the 'classic’ TKRs and 9 (13%) of the 'complete’ TKRs (*p* = 0.03) (Figure 3). For the components of the KSS Knee, no difference between both cohorts in pain (*p* = 0.10), range of motion (*p* = 0.84) and stability (*p* = 0.82) was found. The standard deviation for the KSS Knee is larger in the 'complete’ cohort compared with that in the 'classic’ design cohort (*p* = 0.02) (Table 3).

**Table 3:** Clinical evaluation scores at 5-year follow-up

	**Classic (*n*** **= 72)**	**Complete (*n*** **= 71)**	***p *** **value**
KSS	134.1 ± 38.3	135.0 ± 42.8	0.89
Knee	74.8 ± 17.5	69.7 ± 20.9	0.11
Function	59.2 ± 29.8	65.5 ± 29.6	0.21
OKS	23.3 ±9.3	22.5 ± 10.1	0.45
ROM (degrees)			
Flexion	114.9 ±11.0	115.0 ± 13.6	0.95
Extension	-0.32 ±1.4	-0.92 ± 2.7	0.10

KSS, Knee Society score; OKS, Oxford Knee score; ROM, range of motion. Values are presented as mean ± standard deviation.

No TKR in the 'classic’ cohort had a flexion contracture more than 5° compared with 4 (5.6%) in the 'complete’ cohort (*p* = 0.04). The average KSS Knee of TKRs with a flexion contracture was 52.3 compared with 72.8 in TKRs without a flexion contracture (*p* = 0.04).

In TKRs with reoperation, KSS Knee was 57.8 (SD 25.6) compared with 73.6 (SD 18.3) in TKRs which had no reoperation (*p* = 0.007). The KSS function was 52.1 (SD 25.2) in TKRs with reoperation compared with 63.3 (SD 30.1) in TKRs which had no reoperation (*p* = 0.22). The OKS was 28.0 (SD 10.6) in TKRs with reoperation compared with 22.4 (SD 9.5) in TKRs which had no reoperation (*p* = 0.06).

In TKRs with MUA, KSS Knee was 59.7 (SD 28.1) compared with 72.7 (SD 18.9) in TKRs without MUA (*p* = 0.14). The KSS function was 76.0 (SD 18.2) in patients with MUA compared with 61.8 (SD 30.0) in patients without MUA (*p* = 0.30). The OKS was 21.4 (SD 11.7) in patients with MUA compared with 23.0 (SD 9.6) in patients without MUA (*p* = 0.73).

**Figure 4:**
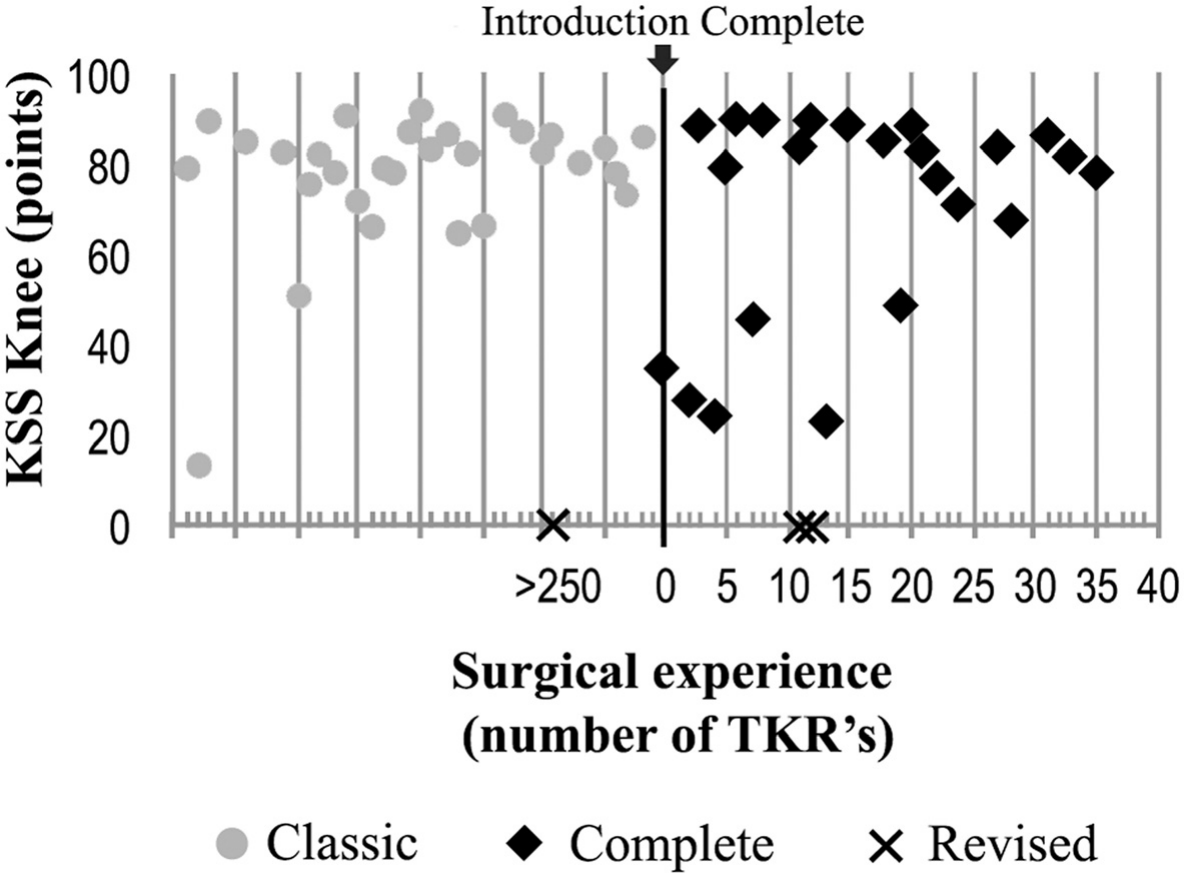
**Series of KSS Knee of the 'classic’ and 'complete’ TKRs performed by a single surgeon.** The surgeon performed >250 TKRs of which 41 were clinically evaluated 'classic’ TKR, before the introduction of the 'complete’ and 37 'complete’ TKRs thereafter. The body of the KKS Knee scores of the 'classic’ design TKRs were in highest three quartiles (>63 points). Of the first 20 'complete’ TKRs, two TKRs were revised and six TKRs scored within the lowest quartile (<63 points).

The learning curve was assessed on TKRs of the senior author, who performed 78 TKRs (55%) of which 41 'classic’ TKRs (52%) and 37 'complete’ TKRs (48%), in this study. In this surgeon's 'classic’ cohort, one TKR (2.4%) was revised compared to two TKRs (5.5%) in this surgeon's 'complete’ cohort (*p* = 0.60) (Figure 4). In this surgeon's 'classic’ cohort, two TKRs (4.8%) scored within the lowest quartile (<63 points) of the KSS Knee compared with six TKR's (16%) in this surgeon's 'complete’ cohort (*p* = 0.14) (Figure 4).

More detailed analysis of the single items of the KSS Knee and additional questions from the Algofunctional Index regarding pain showed no differences except from experienced pain after standing for half an hour (answer options, 'none or scarce’ or 'painful’). In the 'classic’ cohort, 23.6% of TKRs were painful after standing for 30 min compared with 42.3% of the 'complete’ cohort (*p* = 0.02).

## Discussion

No difference between the cohorts could be observed considering the total percentage of revisions, percentage of revisions based on loosening of the tibial tray, average KSS, OKS, flexion and extension. After changing to the 'complete’, a larger percentage of TKRs scored in the lower range of the KSS Knee.

A higher rate of rheumatoid arthritis as underlying diagnosis for TKR was found in the 'classic’ cohort. It is suggested that outcomes in patients with rheumatoid arthritis are likely to be different to patients with OA, owing to a variety of local and systemic effects associated with the disease [13]. In our study, 7 of 182 TKRs (3.8%) placed for osteoarthritis were revised compared to 1 TKR revision of 13 TKRs (7.6%) placed for RA (*p* = 0.43). Therefore, it is not likely that the higher rate of rheumatoid arthritis as underlying diagnosis for TKR in the 'classic’ cohort affects the revision rate of TKRs in this cohort.

Although we did not find a higher revision rate of the 'complete’ compared with the 'classic’, the adjustments to the insert of the 'complete’ might contribute to loosening of the tibial tray. Torque and shear forces in a mobile-bearing TKR will be dissipated from the prosthesis–bone interface by motion of the bearing and by load sharing with soft tissue structures [1,14,15]. By limiting femoral translation by the raised anterior lip of the 'complete’ insert, load sharing with soft tissue is decreased and more momentum is exerted on the tibial tray. Also, the potential rotation of the insert decreases by moving the anchor peg of the tibial component ventrally. These factors combined might increase the stress exerted on the bone–implant interface, which might cause loosening. This hypothesis is consistent with data collected reviewing the indications of all revised LCS TKRs in our hospital, which showed a revision rate of 2.2% in 2,338 LCS TKRs performed from 2001 until 2011. Of all revised 'complete’ TKRs, 80% was due to loosening of the tibial tray and 3.3% due to instability; no revision was performed because of insert dislocation. In case of the 'classic’, 50% was due to loosening of the tibial tray, 5% because of instability and 25% because of insert dislocation.

In order to prevent differences in clinical evaluation, a single independent examiner performed all the clinical assessments. The cohorts showed no differences based on the available patient specific parameters. The pre-, peri- and postoperative management was standardized according to a uniformly applied protocol. This protocol has not been changed during this evaluation. Therefore, it is not likely that postoperative management caused differences in the results.

Through the past years, cemented tibial components have shown less aseptic loosening compared to uncemented tibial components [16]. However, there was no difference in mean KSS between cemented and uncemented tibial components [16]. Because of the low rate of revision due to aseptic loosening with the 'classic’ (1.3%), we did not change to a cemented tibial component during the cohort described in this study.

In this cohort analysis, the KSS Knee is used as a key indicator. Although the mean KSS Knee shows no difference between both cohorts, there was a higher percentage of patients in the 'complete’ cohort scoring in the lower range of the KSS. The KSS Knee is constructed out of pain, range of motion and stability [7]. Persisting pain, limited motion and instability are reasons for revision [17]. Therefore, a higher percentage of patients with a low KSS Knee might result in a higher revision rate.

The broad standard deviation for the 'complete’ cohort may not be fully attributed to the relatively small changes to the design [18]. Surgical technique is a major contributing factor to early revision since early loosening cannot be attributed to polyethylene wear [17]. To support this hypothesis unequivocally, 71 TKRs by three surgeons are not sufficient. A descriptive inquiry of the 'complete’ cohort scores by sorting the KSS Knee of each surgeon's patient in subsequent order may point at a learning curve after the change. The revision rate and the KSS Knee of the TKR's in the 'classic’ and the 'complete’ cohort performed by the orthopaedic surgeon who placed 55% of the TKRs were similar. However, the 2 revisions and the 6 TKRs with KSS Knee scores in the lowest quartile in the 'complete’ cohort can be reduced to the first 20 TKRs. This suggests a learning curve in the first 20 'complete’ TKRs. Unfamiliarity with drilling, the anchoring hole in the tibia may have attributed to this assumed learning curve. Also, the positioning of the tibial tray in AP direction is referenced by the anterior tibial cortex and is not supported by any instrumentation. The outline of the tibial tray has been changed, and its size increased in AP direction. The changes in shape, dimensions and positioning may have affected AP alignment of the tibial tray. A more anterior position leads to increased stress on the patellofemoral joint and less efficient use of the extensor mechanism [19].

Although clinical results of the 'classic’ were satisfactory, changes were made to implant designs, accompanying instrumentation and surgical technique to improve the 'classic’ system. Whether the changes in implant design, accompanying instrumentation and surgical technique between the 'classic’ and the 'complete’ could significantly affect the outcome is unknown. The possibility that relatively minor changes, described as only subtle and often cosmetic [20], could affect outcome was not expected. However, minimal changes in the Insall–Burnstein TKR led to worrying side effects [21]. Surgeons should be cautioned against early adoption of new technologies that have not been proven over time [22,23]. A side effect of an enforced change in design and technique is the necessity to gain experience, and this may lead to unfortunate outcomes until sufficient practical experience has been built up. Transition to the 'complete’ and equipment involves major investments in terms of time and costs. In times of budget constraints, these arguments should be taken into account.

Limitations of this study are mainly due to the retrospective design of this study. Ideally, the introduction of a new prosthesis is preceded by a randomized clinical trial comparing both prostheses with extensive follow-up [24,25]. However, as the 'classic’ was no longer available, a randomized clinical trial was not an option. Second, there are no preoperative knee scores because it was not a standard procedure in our hospital at that time to record these. Although information on preoperative knee flexion and extension was present in a large majority of patients, we decided to exclude this from this study because of probable inaccuracy due to various observers and associated interobserver variability. Third, no radiographic analysis of implant positioning was performed. Although standard AP and lateral views of the clinically evaluated patients at 5 years were present, these X-rays were made as part of clinical follow-up and were not considered accurate enough to assess AP alignment of the tibial tray or the presence of radiolucent lines. Fourth, there was a rather high percentage of patients lost to follow-up. This could be partially attributed to lack of patient's motivation to participate. In a prospective study, these patients are excluded. Finally, we present midterm results at 5 years; therefore, we cannot conclude on possible long-term benefits of the 'complete’.

The sample size was chosen with revision rate as primary outcome measure. A sample size of 100 patients in each cohort with *α* 0.05 and 80% power would have been sufficient to show difference in revision rate when the difference between cohorts was 0%–2% compared with 7%–12%. However, the revision rate in the 'classic’ cohort was 3% compared with 5% in the 'complete’, which results, with *α* 0.05, in 10.8% power. This means that there is only a probability of 10.8% that the 'complete’ cohort has a higher revision rate. With these revision rates, cohorts of 1,500 patients each are needed, which requires a multicentre study.

It is unknown if this disadvantage of changing to a different design is proportional to the improvement on results after solving start-up issues such as learning curve. Satisfactory outcome for the individual patient should be the main interest of a design change. Orthopaedic manufacturers must carefully consider the reasons for introducing new technology as well as the pathway to development and assessment of new TKRs [26].

The perfect knee prosthesis has not been designed yet. Innovations to improve on the existing designs should be encouraged. However, before general introduction of the 'complete’, its advantages should be demonstrated in clinical trials on a limited number of patients and performed by a limited number of surgeons. In this study, there was no difference in 5-year incidence of revision between the 'classic’ and the 'complete’ cohort. Also, there was no difference in midterm clinical performance before and after introduction of the 'complete’. However, a higher percentage of patients with a low KSS knee was found. It is unknown whether this is caused by the change in design and associated surgical technique and instruments or due to the rather high loss to follow-up for clinically evaluated patients (29%) in this study. Further investigation in future studies with the introduction of new designs is needed.

## Abbreviations

AP: anterior posterior; ASA: American Society of Anaesthesiologists; KSS: Knee Society score; LCS: low contact stress; MUA: mobilization under anaesthesia; OKS: Oxford Knee score; TKR: total knee replacement

## Competing interests

The authors declare that they have no competing interests.

## Authors’ contributions

MP, MB and PN made a substantial contribution to the conception and design of the study. MP was responsible for the acquisition of data. All authors contributed to the analysis and interpretation of data. MP and RH were involved in drafting the manuscript; MB and PN critically revised the manuscript for important intellectual content. All authors gave final approval of the version to be published and agreed to be accountable for all aspects of the work in ensuring that questions related to the accuracy or integrity of any part of the work are appropriately investigated and resolved. All authors read and approved the final manuscript.
